# High coverage of targeted lipidomics revealed lipid changes in the follicular fluid of patients with insulin-resistant polycystic ovary syndrome and a positive correlation between plasmalogens and oocyte quality

**DOI:** 10.3389/fendo.2024.1414289

**Published:** 2024-06-06

**Authors:** Meizi Zhang, Yuanyuan Wang, Jianyong Di, Xuanlin Zhang, Ye Liu, Yixin Zhang, Bowen Li, Simeng Qi, Xiaomin Cao, Li Liu, Shouzeng Liu, Fengqin Xu

**Affiliations:** ^1^ Reproductive Medicine Center, Tianjin First Central Hospital, Tianjin, China; ^2^ LipidAll Technologies Company Limited, Changzhou, Jiangsu, China

**Keywords:** polycystic ovary syndrome, follicular fluid, lipidomics, oocyte quality, plasmalogens

## Abstract

**Background:**

Polycystic ovary syndrome with insulin resistance (PCOS-IR) is the most common endocrine and metabolic disease in women of reproductive age, and low fertility in PCOS patients may be associated with oocyte quality; however, the molecular mechanism through which PCOS-IR affects oocyte quality remains unknown.

**Methods:**

A total of 22 women with PCOS-IR and 23 women without polycystic ovary syndrome (control) who underwent *in vitro* fertilization and embryo transfer were recruited, and clinical information pertaining to oocyte quality was analyzed. Lipid components of follicular fluid (FF) were detected using high-coverage targeted lipidomics, which identified 344 lipid species belonging to 19 lipid classes. The exact lipid species associated with oocyte quality were identified.

**Results:**

The number (rate) of two pronuclear (2PN) zygotes, the number (rate) of 2PN cleaved embryos, and the number of high-quality embryos were significantly lower in the PCOS-IR group. A total of 19 individual lipid classes and 344 lipid species were identified and quantified. The concentrations of the 19 lipid species in the normal follicular fluid (control) ranged between 10^-3^ mol/L and 10^-9^ mol/L. In addition, 39 lipid species were significantly reduced in the PCOS-IR group, among which plasmalogens were positively correlated with oocyte quality.

**Conclusions:**

This study measured the levels of various lipids in follicular fluid, identified a significantly altered lipid profile in the FF of PCOS-IR patients, and established a correlation between poor oocyte quality and plasmalogens in PCOS-IR patients. These findings have contributed to the development of plasmalogen replacement therapy to enhance oocyte quality and have improved culture medium formulations for oocyte *in vitro* maturation (IVM).

## Introduction

Polycystic ovary syndrome (PCOS) is the most common endocrine and metabolic disorder in women of reproductive age, with an incidence of 6% to 25% in women of reproductive age (1). PCOS manifests as various metabolic conditions, including obesity, hyperinsulinemia, insulin resistance (IR), and low-grade chronic inflammation. These metabolic disorders not only increase the severity of PCOS but also affect reproductive function. IR accounts for 50% to 70% of PCOS cases caused by metabolic disease (2). PCOS patients account for 30% to 40% of infertile patients. The ESHRE/ASRM guidelines recommend *in vitro* fertilization (IVF) treatment for infertile PCOS patients with sparse ovulation and abnormal ovarian function (3). However, a considerable number of PCOS patients still struggle to conceive even after ovulation induction and IVF treatment. Moreover, PCOS patients typically require a significantly highernumber of IVF treatment cycles compared to non-PCOS patients (4). Studies have suggested that poor fertility in PCOS patients may be related to endometrial receptivity (5) and oocyte quality (6, 7).

Oocyte quality can be assessed by various clinical parameters, including the morphology of oocyte corona cumulus complexes, oocyte maturity, and embryonic developmental potential. In patients with PCOS, adverse fertility outcomes, such as impaired oocyte maturation, decreased fertilization, blastulation, and implantation, as well as increased miscarriage rates, have been observed during different stages of reproduction (7). However, to our knowledge, only a few studies have evaluated the IVF outcomes related to oocyte quality in women with PCOS-IR, and the conclusions and limitations of these studies vary. With the exception of one study where no significant difference was found (8), other studies have suggested that PCOS-IR significantly reduces the number of oocytes retrieved, the number of mature oocytes, the number (rate) of normally fertilized oocytes, and the number (rate) of top-quality embryos (9–12). Moreover, these oocyte quality parameters decrease with increasing Homeostatic Model Assessment of Insulin Resistance (HOMA-IR) in patients with PCOS, suggesting that IR exacerbates adverse clinical outcomes in patients with PCOS (11).

However, the molecular mechanism of poor oocyte quality induced by PCOS-IR remains unclear. IR has been proven to be related to abnormal glucose, amino acid, and lipid metabolism (13). These abnormal metabolites are not only present in plasma but also accumulate abnormally in follicular fluid (FF), which is a complex microenvironment for oocyte growth and development (14). Lipidomics, an important branch of metabolomics, has become a promising technique for examining lipid profiles in body fluids, blood, and tissues. Currently, only a few studies on untargeted lipidomics in women with PCOS have been published (12, 15). However, targeted quantitative lipidomic analysis of FF from PCOS-IR patients has not been well performed. In this study, we used high-coverage targeted lipidomics to analyze lipid concentrations in follicular fluid from non-PCOS women and elucidated the different lipid profiles associated with PCOS-IR. Moreover, this study revealed that the level of plasmalogens was positively correlated with the normal fertilization rate, 2PN cleavage rate, and number of top-quality embryos. These findings could lead to further investigations of the molecular mechanisms of action of plasmalogens in PCOS-IR patients, which could help improve the clinical outcomes of individuals with PCOS-IR.

## Materials and methods

### Ethical approval

This study was approved by the Ethics Boards of Tianjin First Central Hospital (Research License 2019N047KY), and written informed consent was obtained from all participants at enrollment. This study was conducted in accordance with the Declaration of Helsinki (16).

### Subjects and sample collection

The present study was performed at the reproductive medicine center of Tianjin First Central Hospital from November 2019 to May 2021. A total of 45 Chinese Han women of similar age were divided into two groups: 22 PCOS-IR patients and 23 non-PCOS control women. PCOS was diagnosed according to the 2003 Rotterdam criteria, i.e., the presence of two or more cycles of oligo- and/or anovulatory hyperandrogenism, clinical and/or biochemical signs, and polycystic ovaries after the exclusion of other etiologies such as congenital adrenal hyperplasia, androgen-secreting carcinomas, and Cushing’s syndrome. The assessment of insulin resistance was based on the HOMA-IR, which was calculated as fasting insulin (mIU/L) × fasting glucose (mmol/L)/22.5. The control subjects were infertile primarily due to tubal occlusion and endometrial translocations, and these subjects had regular menstrual cycles, normal ovarian morphology, no clinical or biochemical hyperandrogenism, and no insulin resistance or obesity. All subjects can undergo routine IVF.

Grossly clear FF was collected from a single large follicle on the first puncture of each ovary from oocyte retrieval. The FF samples were centrifuged for 10 min at 3,000×g and then stored at -80°C until further use. Fasting blood samples from all the participants were collected on days 2–5 of their natural menstrual cycle or if amenorrhea had occurred for more than 40 days with follicle diameters not exceeding 10 mm for the analysis of levels of sex hormones and some biochemical parameters. Follicle-stimulating hormone (FSH), luteinizing hormone (LH), prolactin (PRL), and testosterone (T) were assessed using enzyme-linked fluorescent assays (VIDAS, Biomérieux, France). The concentration of anti-Müllerian hormone (AMH) was measured using a chemiluminescence immunoassay analyzer (iFlash 3000-H, YHLO, Shenzhen, China). Fasting serum insulin levels were assessed using electrochemiluminescence (cobas e601, Roche Diagnostics, Indianapolis, IN, USA). Fasting glucose levels, total cholesterol (TC), triglycerides (TG), high-density lipoprotein cholesterol (HDL-C), low-density lipoprotein cholesterol (LDL-C), and very low-density lipoprotein (VLDL) were assessed using the enzymatic method with an automated biochemistry analyzer (cobas c701, Roche Diagnostics, Indianapolis, IN, USA) in accordance with stringent quality standards.

### Lipid extraction

Lipids were extracted from FF using a modified version of Bligh and Dyer’s method, as described previously (16). Briefly, 750 µL of chloroform:methanol (1:2, v/v) was added with 10% deionized water. Then, samples were incubated at 1500 rpm for 1 h at 4 °C. At the end of the incubation, 350 µL of deionized water and 250 µL of chloroform were added to induce phase separation. The samples were then centrifuged, and the lower organic phase containing lipids was extracted into a clean tube. Lipid extraction was repeated once by adding 500 µL of chloroform to the remaining tissues in the aqueous phase, and the lipid extracts were pooled into a single tube and dried in a SpeedVac in OH mode. The samples were stored at -80 °C until further analysis.

### Lipidomics analyses

Polar lipids were analyzed using an Exion Ultra Performance Liquid Chromatography (UPLC) system coupled with a triple quadrupole/ion trap mass spectrometer (6500 Plus Qtrap; SCIEX) as described previously (17–20). Separation of individual lipid classes of polar lipids by normal phase High Performance Liquid Chromatography (HPLC) was carried out using a Phenomenex Luna 3 µm-silica column (internal diameter 150 × 2.0 mm) with the following conditions: mobile phase A (chloroform: methanol: ammonium hydroxide, 89.5:10:0.5) and mobile phase B (chloroform: methanol: ammonium hydroxide: water, 55:39:0.5:5.5). Multiple reaction monitoring (MRM) transitions were set up for comparative analysis of various polar lipids. Individual lipid species were quantified by referencing spiked internal standards. Dimyristoyl phosphatidylglycerol (DMPG), C14- lysobisphosphatidic acid (LBPA), dimyristoyl phosphatidylcholine (DMPC), dimyristoyl phosphatidylethanolamine (DMPE), C12-sphingomyelins (SM), dic8-phosphatidylinositols (PI), monosialodihexosyl gangliosides (GM3)-d18:1/18:0-d3, C17-phosphatidic acids (PA), C17:0-ysophosphatidylcholine (LPC), C17:0-lysophosphatidic acid (LPA), and C17:1-lysophosphatidylinositols (LPI) were obtained from Avanti Polar Lipids. Glycerol lipids, including diacylglycerols (DAGs) and triacylglycerols (TAGs), were quantified using a modified version of reversed-phase HPLC/MRM. The separation of neutral lipids was achieved on a Phenomenex Kinetex-C18 2.6 µm column (i.d. 4.6x100 mm) using an isocratic mobile phase conprising chloroform: methanol: 0.1 M ammonium acetate 100:100:4 (v/v/v) at a flow rate of 170 µL for 17 min. The levels of short-, medium-, and long-chain TAGs were calculated by referencing spiked internal standards of TAG (14:0)3-d5, TAG (16:0)3-d5, and TAG (18:0)3-d5 obtained from CDN isotopes, respectively. DAGs were quantified using d5-DAG17:0/17:0 and d5-DAG18:1/18:1 as internal standards (Avanti Polar Lipids). Free cholesterols and cholesteryl esters were as described previously, with d6-cholesterol and d6-C18:0 cholesteryl ester (CE) (CDN isotopes) serving as internal standards. Free fatty acids were quantitated using d31-16:0 (Sigma-Aldrich) and d8-20:4 (Cayman Chemicals) as internal standards.

### Statistical analyses

The significant differences between the PCOS-IR and non-PCOS FF samples were determined using the Student’s t-test, the chi-square test, or the Mann-Whitney test. Correlations were assessed by Pearson’s correlation coefficient. The results were statistically significant at p < 0.05.

## Results

### Baseline characteristics of patients and ART outcomes

We collected FF samples from 22 PCOS-IR patients and 23 non-PCOS controls. The baseline characteristics of the PCOS-IR and control groups are summarized in **Table 1**. These two groups were similar in age. However, the women in the PCOS-IR group had significantly higher BMIs, fasting glucose levels, insulin levels, and HOMA-IR scores than those in the control group. Compared with the control group, the PCOS-IR group exhibited a broad spectrum of metabolic and endocrine changes, such as significantly elevated TG levels, AMH, and testosterone, an increased LH to FSH ratio and notably decreased FSH, and PRL levels. These changes were consistent with the clinical phenotypes of PCOS-IR. In addition, we analyzed the assisted reproductive technology (ART) outcomes from IVF cycles in the PCOS-IR and control groups (**Table 1**). There were no significant differences in the total dose of gonadotrophins, peak estradiol levels, number of oocytes retrieved, number of fertilized oocytes, number of transferable embryos, and clinical pregnancy rate between the PCOS-IR and control groups. The number (rate) of 2PN zygotes, the number (rate) of 2PN cleavage, and the number of top-quality embryos were significantly lower in women with PCOS-IR.

**Table 1:** The clinical parameters in control and PCOS patients.

Parameters	Control	PCOS	p value	test
**n**	23	22		
Baseline Characteristics				
**Age (years)**	33 (26-39)	30 (22-39)	ns	^a^
**BMI (kg/m2)**	21.53 (2.37)	23.20 (2.24)	**0.019**	^b^
**FSH (mIU/mL)**	6.20 (1.74)	5.05 (1.69)	**0.03**	^b^
**LH (mIU/mL)**	4.02 (1.67)	5.25 (3.55)	ns	^b^
**LH/FSH**	0.65 (0.21)	1.17 (0.91)	**0.01**	^b^
**Testosterone (ng/mL)**	0.32 (0.14)	0.44 (0.19)	**0.022**	^b^
**Prolactin (ng/mL)**	21.17 (9.86)	16.09 (6.49)	**0.048**	^b^
**AMH (ng/mL)**	3.85 (1.67)	9.17 (5.38)	**<0.001**	^b^
**Fasting Glucose (mM)**	4.84 (0.36)	5.31 (0.30)	**<0.001**	^b^
**Fasting Insulin (μIU/mL)**	5.47 (1.45)	17.02 (9.26)	**<0.001**	^b^
**HOMA-IR**	1.17 (0.31)	4.02 (2.22)	**<0.001**	^b^
**TC (mM)**	4.45 (0.63)	4.39 (1.08)	ns	^b^
**TG (mM)**	0.90 (0.31)	1.58 (1.18)	**0.011**	^b^
**HDL-C (mM)**	1.46 (0.25)	1.34 (0.32)	ns	^b^
**LDL-C (mM)**	2.79 (0.59)	2.89 (0.74)	ns	^b^
**VLDL (mM)**	0.15 (0.09)	0.18 (0.22)	ns	^b^
ART outcomes				
**Total dose of gonadotrophins (IU)**	2674.24 (858.40)	2540.45 (1539.32)	ns	^b^
**Peak Estradiol (pg/mL)**	4815.52 (1935.51)	4799.27 (2071.00)	ns	^b^
**Oocytes retrieved (n)**	15.00 (4.34)	13.45 (3.70)	ns	^b^
**Fertilized oocytes (n)**	13.04 (4.42)	11.18 (3.61)	ns	^b^
**Fertilized oocytes (%)**	86.90 (12.96)	82.72 (10.68)	ns	^b^
**2PN (n)**	12.26 (4.00)	9.23 (3.05)	**0.007**	^b^
**2PN (%)**	82.17 (13.12)	68.87 (15.46)	**0.003**	^b^
**2PN cleavage embryos (n)**	12.09 (4.06)	9.18 (3.06)	**0.01**	^b^
**2PN cleavage embryos (%)**	93.07 (6.80)	82.54 (13.37)	**0.002**	^b^
**transferable embryos (n)**	6.22 (2.56)	5.32 (2.19)	ns	^b^
**transferable embryos (%)**	53.48 (20.34)	62.15 (27.22)	ns	^b^
**Top-quality embryos (n)**	5.09 (3.06)	3.36 (2.08)	**0.033**	^b^
**Top-quality embryos (%)**	43.19 (21.97)	40.19 (26.23)	ns	^b^
**Clinical pregnancy (%)**	22 (12)	21 (8)	ns	^c^

p-values > 0.05 were considered non-significant (ns). ^a^, two-sample Mann-Whitney test. Values are median (minimum–maximum). ^b^, two-sample t-test. Values are the mean (SD). ^c^, Chi-square test. Values are counts.

### Concentrations of individual lipid classes in non-PCOS follicle (control) fluid

**Figure 1:**
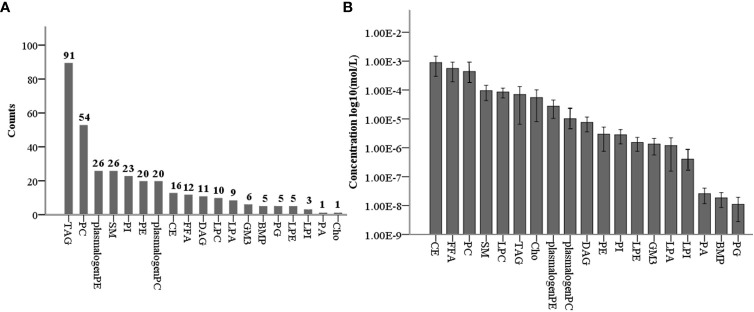
Levels of 19 lipid classes in normal non-PCOS follicular fluid. **(A)** Counts of 344 lipid species in 19 lipid classes for targeted lipidomic analysis. **(B)** Concentration profiles of 19 lipid classes in normal non-PCOS follicular fluid.

Using targeted lipidomics technology, we identified and quantified a total of 344 lipid species of 19 lipid classes (**Figure 1A**), which included cholesterol (Cho, 1), CE (16), free fatty acids (FFA, 13), TAG(91), DAG (11), phosphatidylcholines (PC, 54), PC plasmalogens (20), phosphatidylethanolamines (PE, 20), PE plasmalogens (20), PI (23), phosphatidylglycerols (PG, 5), PA (1), LPC (10), lysophosphatidylethanolamine (LPE, 5), LPA (9), LPI (3), bis(monoacylglycerol)phosphate (BMP, 5), SM (26), and GM3 (6) (**Figure 1A**). To analyze the profile of 19 lipid classes in follicle fluid, follicle fluid samples from 23 non-PCOS patients were quantitatively measured. The results showed that the level of CE was the highest (8.93E^-4^ ± 2.97E^-4^ mol/L), followed by that of FFA, PC, SM, LPC, TAG, Cho, and plasmalogen PE, and that of PG was the lowest (1.10E^-8^ ± 4.12E^-9^ mol/L) (**Figure 1B**).

### Identification of significantly altered lipid molecular species in PCOS-IR follicle fluid

**Figure 2:**
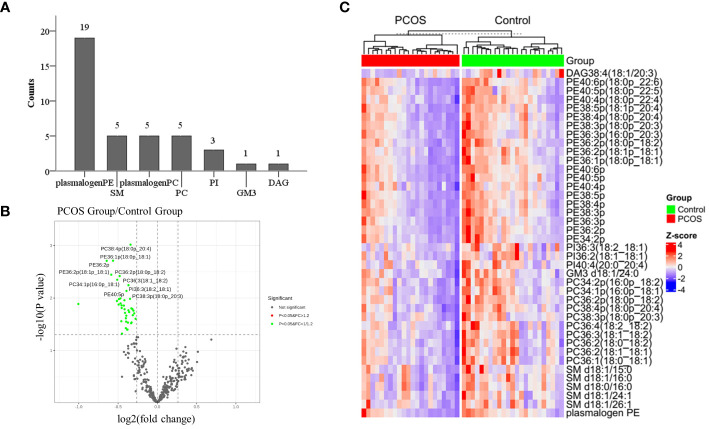
Identification and bioinformatics analysis of significantly differential lipid species. **(A)** Thirty-nine lipid species with significant differences were distributed in 7 lipid classes, such as PE/PC plasmalogen, SM, and PC. **(B)** Volcano plot showing statistical significance (p values) and fold changes for 344 lipids. **(C)** Heatmap of 39 significantly differentially abundant lipids.

**Figure 3:**
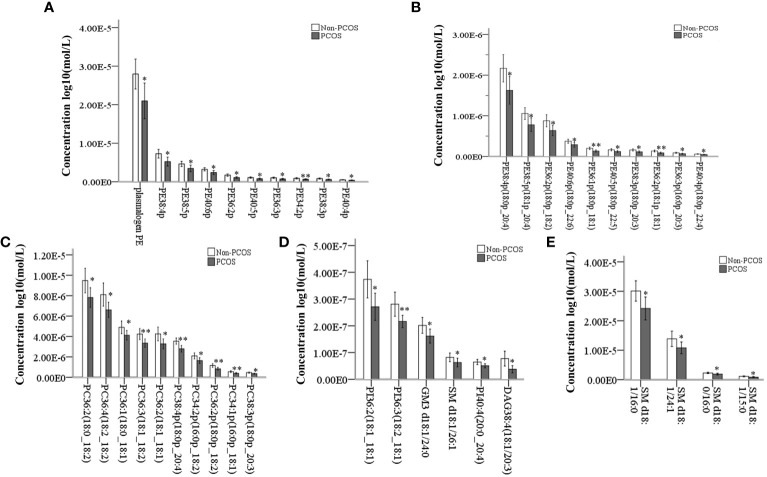
Quantitative analysis of significantly different lipid species between PCOS-IR and non-PCOS follicular fluid samples. **(A, B)** 19 significantly altered PE plasmalogens. **(C)** Significantly altered PC and PC plasmalogens. **(D)** Patients with significantly altered PE, PI, and GM3. **(E)** Patients with significantly altered SM. *P < 0.05, **P < 0.01. Error bars show the standard deviation.

Out of the 344 lipids analyzed, 39 showed significant differences between the study and control groups. These lipids included PE plasmalogens, PC plasmalogens, SM, PC, PI, GM3, and DAG, with PE plasmalogens being the most abundant (**Figure 2A**). The distribution of p-values for differential lipids is shown in **Figure 2B**. Furthermore, significantly different lipid concentrations in 22 PCOS-IR patients and 23 control patients were normalized to Z values and visualized in a heatmap (**Figure 2C**). Plasmalogens are distributed in various membrane systems and play important roles in many biological functions. The level of PE plasmalogens predominated over the level of plasmalogen PC in most tissues (21). In this study, the concentrations of 19 PE plasmalogens in PCOS-IR follicular fluid were lower than those in the control group(p < 0.05) (**Figures 3A, B**), and the concentrations of five PC plasmalogens molecules were significantly lower in PCOS-IR follicular fluid than in the control group(p < 0.05) (**Figure 3C**). SM, the primary sphingomyelin in mammalian cell membranes, interacts with cholesterol, and this interaction has numerous significant functional consequences (22). In this study, twenty-six SM species were detected, and the concentrations of five SM molecules in PCOS-IR follicular fluid were lower than those in the control group (p < 0.05) (**Figures 3D, E**). PC is the primary phospholipid on the surface monolayer of all lipoproteins, and the concentrations of five PC molecules in PCOS-IR follicular fluids were significantly lower than those in the control group (p < 0.05) (**Figure 3C**). In addition, the concentrations of three PIs, one GM3, and one DAG in the PCOS-IR group were significantly lower than those in the control group (p < 0.05) (**Figure 3D**).

### The level of plasmalogens in follicular fluid was positively correlated with oocyte quality

The above study demonstrated that the number (rate) of 2PN zygotes, the number (rate) of 2PN cleavage embryos, and the number of top-quality embryos, which are related to oocyte quality, were significantly lower in the PCOS-IR group compared to the control group (**Table 1**). To further assess whether the changes in individual lipid classes affect the levels of these indicators related to oocyte quality, we analyzed the relationships between lipid levels and these indicators in 45 patients. Among the 19 significantly altered PE plasmalogens, 13 were positively correlated with the number of top-quality embryos. These include PE40:6p (18:0p_22:6), PE40:5p (18:0p_22:5), PE38:5p(18:1p_20:4), PE36:2p(18:0p_18:2), PE36:2p(18:1p_18:1), PE36:1p(18:0p_18:1), PE40:6p, PE40:5p, PE40:4p, PE38:5p, PE36:3p, PE36:2p, and PE34: 2p. Additionally, the 2PN cleavage rate showed a positive correlation with two PE plasmalogens, PE40:6p (18:0p_22:6) and PE40:5p., Furthermore, the PE plasmalogen that was positively correlated with the 2PN rate was PE40:5p. In addition, the only PC plasmalogen that was positively correlated with the 2PN number and 2PN cleavage number was PC38:4p (18:0p_20:4) (**Table 2**).

**Table 2:** Correlation of 39 lipid species in follicle liquid samples with significantly lower oocyte quality parameters (n = 45).

	Lipid species	Pearson correlation coefficient	P value
2PN	PC38:4p(18:0p_20:4)	0.299	**0.046**
	DAG38:4(18:1/20:3)	0.38	**0.01**
2PN Rate	PE40:5p	0.302	**0.044**
2PN Cleavage Embryos	DAG38:4(18:1/20:3)	0.385	**0.009**
	PC38:4p(18:0p_20:4)	0.294	**0.05**
2PN Cleavage Rate	PE40:6p(18:0p_22:6)	0.329	**0.028**
	PE40:5p	0.303	**0.043**
Top-quality Embryos	total plasmalogen PE	0.308	**0.04**
	PE40:6p(18:0p_22:6)	0.35	**0.018**
	PE40:5p(18:0p_22:5)	0.327	**0.028**
	PE38:5p(18:1p_20:4)	0.34	**0.022**
	PE36:2p(18:0p_18:2)	0.384	**0.009**
	PE36:2p(18:1p_18:1)	0.348	**0.019**
	PE36:1p(18:0p_18:1)	0.337	**0.023**
	PE40:6p	0.351	**0.018**
	PE40:5p	0.332	**0.026**
	PE40:4p	0.341	**0.022**
	PE38:5p	0.308	**0.039**
	PE36:3p	0.31	**0.038**
	PE36:2p	0.309	**0.039**
	PE34:2p	0.305	**0.042**

## Discussion

In this case-control study of 22 women with PCOS-IR and 23 women without PCOS, the number (rate) of 2PN zygotes, the number (rate) of 2PN cleaved embryos, and the number of high-quality embryos, which are associated with oocyte quality, were significantly lower in the PCOS-IR group. High-coverage targeted lipidomic analysis of the follicular fluid revealed that 19 lipid classes in the non-PCOS group had concentrations ranging from 10^-3^ to 10^-9^ mol/L., The most abundant lipid class was CE, while the lease abundant class was PG. Fifty-nine lipids were significantly reduced in the PCOS-IR group, with PE being the most abundant lipid species, followed by the other PC plasmalogens, SM, PC, PI, GM3, and DAG. The correlations between these lipids and oocyte quality-related indicators showed that the presence of plasmalogens positively correlated with oocyte quality or competence.

Lipidomic analysis technology is generally based on liquid chromatography-mass spectrometry (LC-MS) platforms and can be divided into nontargeted and targeted analyses. Non-targeted lipidomics can be used to systematically analyze various types of lipids in a sample without bias, while targeted lipidomics is primarily employed for selective and specific quantitative analysis of specific lipids (15). Advances in targeted lipidomics have made it possible to quantitatively analyze the entire lipid pool of biological samples. This study is the first to use a high-coverage targeted lipidomics technique to quantify lipids in five categories and 19 classes of follicular fluid from non-PCOS women. The fatty acyls(FFA), glycerolipids (DAG, TAG), glycerophospholipids (PC, PC plasmalogen, PE plasmalogen, LPC, PE, LPE, PG, BMP, PI, LPI, PA, LPA), sphingolipids (SM, GM3), and sterol lipids (Cho, CE) were detected. Among the 344 lipids, CE18:2 had the highest level(4.6E^-3^ ± 1.2E^-3^ mol/L), and PG38:6 (18:2:4) had the lowest level (1.26E^-9^ ± 5.25E^-10^ mol/L) (unpublished). The non-PCOS group excluded women who had irregular menstruation, abnormal ovarian morphology, hyperandrogenism, insulin resistance or hyperinsulinemia, and obesity. Therefore, the lipid levels in non-PCOS follicular fluid may represent those in normal follicular fluid. These determined lipid concentrations can serve as a reference to enhance the culture system for oocyte IVMand embryonic development.

Plasmalogens are a lipid subclass characterized by a vinyl ether-bonded aliphatic group attached to the sn-1 position of glycerol, a fatty acid esterified at the sn-2 position, and typically a phosphatidylethanolamine or phosphatidylcholine attached to the sn-3 position. The sn-2 fatty acids of plasmalogens are usually enriched with polyunsaturated fatty acids such as arachidonic acid (20:4) or docosahexaenoic acid (22:6). The steady-state level of plasmalogens is determined by the balance between the biosynthesis rate and degradation rate. The biosynthesis of plasmalogens begins in the peroxisome and ends in the endoplasmic reticulum (ER) (23). In the peroxisome, plasmalogen biosynthesis begins with dihydroxyacetone phosphate (DHAP), which undergoes three sequential reactions to generate 1-alkyl-2-lyso-sn-glycerol-3-phosphate (AGP). AGP is transported to the ER, where final biochemical reactions occur. Fatty acyl-CoA reductase 1 (Far1), an enzyme that binds to peroxisomes, is the rate-limiting enzyme in the biosynthesis of plasmalogens (24). Plasmalogen degradation can occur through either non-enzymatic or enzymatic biochemical reactions. The mechanism of nonenzymatic plasmalogen degradation is chemical in nature and depends on the oxidation or hydrolysis of the vinyl-ether bond, i.e., removal of the alkyl chain at the sn-1 position of the glycerol molecule by free radical or acid attack (25). The enzymatic mechanism mainly depends mainly on the action of phospholipases, each of which may have different substrate specificities. Plasmalogens may be involved in the pathophysiological processes of hypoxia, inflammation, oxidative stress, and ferroptosis in patients with PCOS.

In addition to atmospheric oxygen tension, developing and postovulatory ruptured follicles are in a hypoxic state that is thought to be maintained by the hypoxia-inducible factor-1alpha (HIF-1α) signaling pathway expressed by granulosa cells (26). However, women with PCOS have reduced levels of HIF-1α and its target gene, indicating that the hypoxic state of granulosa cells is disrupted in PCOS patients (27). It has been shown that plasmalogen biosynthesis is increased in hypoxia. The genes involved in plasmalogen synthesis, such as glyceronephosphate O-acyltransferase (*GNPAT*), alkylglycerone phosphate synthase (*AGPS*), FAR1/2, and transmembrane protein 189 (*TMEM189*), are also essential for growth under hypoxic conditions (28). Therefore, we speculate that the disturbed hypoxic status of granulosa cells in PCOS patients may disrupt plasmalogen biosynthesis and reduce the level of plasmalogens, which is consistent with our findings.

GCs from patients with PCOS show clear signs of inflammation and oxidative stress, resulting in a significant decrease in proliferation and an increase in apoptosis (29, 30). In this study, we found that the plasminogen levels in the follicular fluid of patients with PCOS were significantly lower than those in the follicular fluid of control group. Plasminogens and their metabolites are known to possess antioxidant potential and immunomodulatory effects. This is due to the increased electron density of the vinyl ether bond at the sn-1 position, which enhances susceptibility to cleavage bt reactive oxygen species (ROS) (31). Polyunsaturated fatty acids (PUFAs), especially docosahexaenoic acid and arachidonic acid, located at the sn-2 position, can be released by phospholipase A2 (PLA2)., Arachidonic acid can then be further metabolized by cyclooxygenases, lipoxygenases, and cytochrome P450 enzymes to produce a variety of bioactive mediators., These include prostaglandins, leukotrienes, eicosatrienoic acids, dihydroxyeicosatetraenoic acids, eicosatetraenoic acids, and lipotoxins, which play a crucial role in immunomodulatory functions (32). Docosahexaenoic acid is a precursor of the anti-inflammatory lipid mediators resolvins and protectins (33–35). However, it remains to be further determined whether these metabolic pathways involving plasmalogens are involved in the pathophysiological processes of PCOS. It has been shown that AA (C20:4n6) levels are higher in PCOS patients compared to healthy controls, and AA may induce oxidative stress (OS) and upregulate the expression of growth differentiation factor 15 in human ovarian granulosa tumor cell lines (KGN) (36).

Ferroptosis is a form of regulated cell death characterized by iron-dependent phospholipid (PL) peroxidation. The substrates of PL peroxidation in ferroptosis are PLs containing PUFA chains at the sn2 position, which are presumably largely plasmalogens (37). Ferroptosis contributes to cellular and tissue damage in various human diseases, such as cancer, cardiovascular diseases, neurodegeneration, liver disease, and ovarian diseases. The pathogenesis of PCOS may be related to the ferroptosis of granulosa cells. Previous studies have shown that ferroptosis in patients with PCOS is regulated by iron homeostasis, redox balance, lipid metabolism, and glutathione metabolism (38–47). However, studies on the PL substrate have not yet been reported. This study revealed that follicular plasmalogen levels were reduced in PCOS patients, suggesting that plasmalogens might also be involved in the ferroptosis of granulosa cells as a substrate for PL peroxidation. Plasmalogens are essential for neuronal cell survival and neuron excitability regulation through the ERK and AKT signaling pathways in Alzheimer’s and Parkinson’s diseases (48, 49). Eicosapentaenoic acid-enriched ethanolamine plasmalogen improved learning and memory deficits by inhibiting neuronal apoptosis and enhancing the brain-derived neurotrophic factor (BDNF)/tropomyosin receptor kinase B (TrkB)/cAMP response element-binding protein (CREB) signaling pathway in cultured cells and mice (50, 51). Plasmalogens also accelerate hair growth and enhance the phosphorylation of AMP-activated protein kinase (AMPK) by stimulating the activity of transient receptor potential cation channel subfamily C member 4 in fibroblasts and hair follicles (52). These findings suggest a potential role of plasmalogens in regulating signaling pathways. However, it is necessary to further investigate whether these signaling pathways involving plasmalogens are implicated in the pathological process of PCOS-IR.

This study found that the level of plasmalogens in the follicular fluid of PCOS-IR patients was significantly lower than that of the control group. It was hypothesized that plasmalogens may be involved in the pathophysiological processes of hypoxia, inflammation, oxidative stress, and ferroptosis in the granulosa cells (GC) of PCOS patients. The disturbed hypoxia in the GC of patients with PCOS may disrupt plasmalogen biosynthesis and reduce the levels of plasmalogens. The granulosa cells of patients with PCOS exhibit noticeable inflammation and oxidative stress, potentially involving catabolic metabolites of plasmalogens such as docosahexaenoic acid and arachidonic acid. Plasmalogens are major substrates for ferroptosis, but the role of plasmalogens in the ferroptosis of granulosa cells in PCOS patients has not been reported. All of the aforementioned pathophysiological processes can result in abnormal granulosa cell function and impact oocyte quality in patients with PCOS. However, the molecular mechanisms associated with plasmalogens need to be further investigated.

It should be noted that this study had some limitations. Firstly, the follicular fluid (FF) analyzed in this study is not fully representative of its natural state because this FF was collected from patients undergoing gonadotropin stimulation. Therefore, the concentrations of various lipids in “normal” follicular fluid may be skewed. Secondly, the dysregulation of plasmalogens enrichment observed in PCOS patients awaits further mechanistic validation. Our preliminary evidence suggests that the decreases in plasmalogens might be associated with poor oocyte quality. Furthermore, we used 22 PCOS-IR and 23 control participants to elucidate the differences in lipid composition between the two groups. However, the relatively small sample size could impact the statistical power of the analysis.

## Conclusions

In conclusion, our study presented the first comprehensive and quantitative analysis of the lipid composition of human follicular fluid using high-coverage targeted lipidomics. This information is valuable for enhancing oocyte *in vitro* maturation culture systems. By comparing the differences in lipid content between normal and PCOS-IR follicular fluid, we identified several unique lipid classes and lipid molecular species associated with PCOS-IR. This discovery opens up new possibilities for further mechanistic studies. To our knowledge, this is the first study to establish a correlation between oocyte quality and plasmalogen levels. Plasmalogens are anticipated to serve as a novel biomarker for the diagnosing and treating of infertility in women with PCOS-IR.

## Data availability statement

The original contributions presented in the study are included in the article/supplementary material. Further inquiries can be directed to the corresponding authors.

## Ethics statement

The studies involving humans were approved by Medical Ethics Committee of Tianjin First Central Hospital. The studies were conducted in accordance with the local legislation and institutional requirements. The participants provided their written informed consent to participate in this study.

## Author contributions

MZ: Funding acquisition, Investigation, Methodology, Project administration, Writing – original draft, Writing – review & editing. YW: Resources, Writing – original draft. JD: Resources, Writing – review & editing. XZ: Resources, Writing – original draft. YL: Investigation, Methodology, Writing – original draft. YZ: Resources, Writing – original draft. BL: Data curation, Writing – original draft. SQ: Methodology, Writing – original draft. XC: Investigation, Writing – review & editing. LL: Supervision, Writing – review & editing. SL: Data curation, Writing – review & editing. FX: Project administration, Supervision, Writing – review & editing.

## References

[1] Cordeiro FB Cataldi TR Do VTDC de Lima CB Stevanato J Zylbersztejn DS. Follicular fluid lipid fingerprinting from women with PCOS and hyper response during IVF treatment. J Assist Reprod Genet. (2015) 32:45–54. doi: 10.1007/s10815-014-0375-0 25374394 PMC4294864

[2] Legro RS Castracane VD Kauffman RP. Detecting insulin resistance in polycystic ovary syndrome: purposes and pitfalls. Obstet Gynecol Surv. (2004) 59:141–54. doi: 10.1097/01.OGX.0000109523.25076.E2 14752302

[3] ESHRE/ASRM-Sponsored PCOS Consensus Workshop Group. Consensus on infertility treatment related to polycystic ovary syndrome. Hum Reprod. (2008) 23:462–77. doi: 10.1093/humrep/dem426 18308833

[4] Hart R Doherty DA. The potential implications of a PCOS diagnosis on a woman's long-term health using data linkage. J Clin Endocrinol Metab. (2015) 100:911–9. doi: 10.1210/jc.2014-3886 25532045

[5] Piltonen TT. Polycystic ovary syndrome: Endometrial markers. Best Pract Res Clin Obstet Gynaecol. (2016) 37:66–79. doi: 10.1016/j.bpobgyn.2016.03.008 27156350

[6] Qiao J Feng HL. Extra- and intra-ovarian factors in polycystic ovary syndrome: impact on oocyte maturation and embryo developmental competence. Hum Reprod Update. (2011) 17:17–33. doi: 10.1093/humupd/dmq032 20639519 PMC3001338

[7] Palomba S Daolio J La Sala GB. Oocyte competence in women with polycystic ovary syndrome. Trends Endocrinol Metab. (2017) 28:186–98. doi: 10.1016/j.tem.2016.11.008 27988256

[8] Cakiroglu Y. The impact of body mass index and insulin resistance on IVF outcomes of polycystic ovary syndrome. North Clin Istanbul. (2017) 4:218–24. doi: 10.14744/nci.2017.79663 PMC5724915 29270569

[9] Hassani F Oryan S Eftekhari-Yazdi P Bazrgar M Moini A Nasiri N. Association between the number of retrieved mature oocytes and insulin resistance or sensitivity in infertile women with polycystic ovary syndrome. Int J Fertil Steril. (2019) 12:310–5. doi: 10.22074/ijfs.2019.5422 PMC6186286 30291692

[10] Naigaonkar A Dadachanji R Hinduja I Mukherjee S. Altered redox status may contribute to aberrant folliculogenesis and poor reproductive outcomes in women with polycystic ovary syndrome. J Assist Reprod Genet. (2021) 38:2609–23. doi: 10.1007/s10815-021-02241-x PMC8581097 34041658

[11] Chen Y Guo J Zhang Q Zhang C. Insulin resistance is a risk factor for early miscarriage and macrosomia in patients with polycystic ovary syndrome from the first embryo transfer cycle: A retrospective cohort study. Front Endocrinol (Lausanne). (2022) 13:853473. doi: 10.3389/fendo.2022.853473 35498421 PMC9046670

[12] Ding Y Jiang Y Zhu M Zhu Q He Y Lu Y. Follicular fluid lipidomic profiling reveals potential biomarkers of polycystic ovary syndrome: A pilot study. Front Endocrinol (Lausanne). (2022) 13:960274. doi: 10.3389/fendo.2022.960274 36176459 PMC9513192

[13] Trevisan R Nosadini R Avogaro A Lippe G Duner E Fioretto P. Type I diabetes is characterized by insulin resistance not only with regard to glucose, but also to lipid and amino acid metabolism. J Clin Endocrinol Metab. (1986) 62:1155–62. doi: 10.1210/jcem-62-6-1155 3517029

[14] Bayasula Iwase A Kobayashi H Goto M Nakahara T Nakamura T. A proteomic analysis of human follicular fluid: comparison between fertilized oocytes and non-fertilized oocytes in the same patient. J Assist Reprod Genet. (2013) 30:1231–8. doi: 10.1007/s10815-013-0004-3 PMC3800524 23888310

[15] Ban Y Ran H Chen Y Ma L. Lipidomics analysis of human follicular fluid form normal-weight patients with polycystic ovary syndrome: a pilot study. J Ovarian Res. (2021) 14:135. doi: 10.1186/s13048-021-00885-y 34645507 PMC8515674

[16] World Medical Association. World Medical Association Declaration of Helsinki: ethical principles for medical research involving human subjects. JAMA. (2013) 310:2191–4. doi: 10.1001/jama.2013.281053 24141714

[17] Lam SM Zhang C Wang Z Ni Z Zhang S Yang S. A multi-omics investigation of the composition and function of extracellular vesicles along the temporal trajectory of COVID-19. Nat Metab. (2021) 3:909–22. doi: 10.1038/s42255-021-00425-4 34158670

[18] Lu J Lam SM Wan Q Shi L Huo Y Chen L. High-coverage targeted lipidomics reveals novel serum lipid predictors and lipid pathway dysregulation antecedent to type 2 diabetes onset in normoglycemic chinese adults. Diabetes Care. (2019) 42:2117–26. doi: 10.2337/dc19-0100 31455687

[19] Lam SM Wang R Miao H Li B Shui G. An integrated method for direct interrogation of sphingolipidhomeostasis in the heart and brain tissues of mice through postnataldevelopment up to reproductive senescence. Analytica Chimica Acta. (2018) 1037:152–8. doi: 10.1016/j.aca.2018.01.015 30292289

[20] Lam SM Wang Z Li J Huang X Shui G. Sequestrationof polyunsaturated fatty acids in membrane phospholipids of Caenorhabditis elegans dauer larvaattenuates eicosanoid biosynthesis for prolonged survival. Redox Biol. (2017) 12:967–77. doi: 10.1016/j.redox.2017.05.002 PMC5429230 28499251

[21] Messias MCF Mecatti GC Priolli DG de Oliveira Carvalho P. Plasmalogen lipids: functional mechanism and their involvement in gastrointestinal cancer. Lipids Health Dis. (2018) 17:41. doi: 10.1186/s12944-018-0685-9 29514688 PMC5842581

[22] Slotte JP. Biological functions of sphingomyelins. Prog Lipid Res. (2013) 52:424–37. doi: 10.1016/j.plipres.2013.05.001 23684760

[23] Nagan N Zoeller RA. Plasmalogens: biosynthesis and functions. Prog Lipid Res. (2001) 40:199–229. doi: 10.1016/S0163-7827(01)00003-0 11275267

[24] Honsho M Asaoku S Fujiki Y. Posttranslational regulation of fatty Acyl-CoA reductase 1, Far1, controls ether glycerophospholipid synthesis. J Biol Chem. (2010) 285:8537–42. doi: 10.1074/jbc.M109.083311 PMC2838275 20071337

[25] Gorgas K Teigler A Komljenovic D Just WW. The ether lipid-deficient mouse: tracking down plasmalogen functions. Biochim Biophys Acta. (2006) 1763:1511–26. doi: 10.1016/j.bbamcr.2006.08.038 17027098

[26] Tang Z Xu R Zhang Z Shi C Zhang Y Yang H. HIF-1α Protects granulosa cells from hypoxia-induced apoptosis during follicular development by inducing autophagy. Front Cell Dev Biol. (2021) 9:631016. doi: 10.3389/fcell.2021.631016 33553188 PMC7862574

[27] Szymanska M Shrestha K Girsh E Harlev A Eisenberg I Imbar T. Reduced endothelin-2 and hypoxic signaling pathways in granulosa-lutein cells of PCOS women. Int J Mol Sci. (2021) 22:8216. doi: 10.3390/ijms22158216 34360981 PMC8347025

[28] Jain IH Calvo SE Markhard AL Skinner OS To TL Ast T. Genetic screen for cell fitness in high or low oxygen highlights mitochondrial and lipid metabolism. Cell. (2020) 181:716–27. doi: 10.1016/j.cell.2020.03.029 PMC7293541 32259488

[29] Lai Q Xiang W Li Q Zhang H Li Y Zhu G. Oxidative stress in granulosa cells contributes to poor oocyte quality and IVF-ET outcomes in women with polycystic ovary syndrome. Front Med. (2018) 12:518–24. doi: 10.1007/s11684-017-0575-y 29260383

[30] Sak S Uyanikoglu H Incebiyik A Incebiyik H Hilali NG Sabuncu T. Associations of serum fetuin-A and oxidative stress parameters with polycystic ovary syndrome. Clin Exp Reprod Med. (2018) 45:116–21. doi: 10.5653/cerm.2018.45.3.116 PMC6125147 30202741

[31] Lessig J Fuchs B. Plasmalogens in biological systems: their role in oxidative processes in biological membranes, their contribution to pathological processes and aging and plasmalogen analysis. Curr Med Chem. (2009) 16:2021–41. doi: 10.2174/092986709788682164 19519379

[32] Wallner S Schmitz G. Plasmalogens the neglected regulatory and scavenging lipid species. Chem Phys Lipids. (2011) 164:573–89. doi: 10.1016/j.chemphyslip.2011.06.008 21723266

[33] Schwab JM Chiang N Arita M Serhan CN. Resolvin E1 and protectin D1 activate inflammation-resolution programmes. Nature. (2007) 447:869–74. doi: 10.1038/nature05877 PMC2757086 17568749

[34] Ariel A Serhan CN. Resolvins and protectins in the termination program of acute inflammation. Trends Immunol. (2007) 28:176–83. doi: 10.1016/j.it.2007.02.007 17337246

[35] González-Périz A Horrillo R Ferré N Gronert K Dong B Morán-Salvador E. Obesity-induced insulin resistance and hepatic steatosis are alleviated by omega-3 fatty acids: a role for resolvins and protectins. FASEB J. (2009) 23:1946–57. 10.1096/fj.08-125674 PMC2698663 19211925

[36] Ma Y Zheng L Wang Y Gao Y Xu Y. Arachidonic acid in follicular fluid of PCOS induces oxidative stress in a human ovarian granulosa tumor cell line (KGN) and upregulates GDF15 expression as a response. Front Endocrinol (Lausanne). (2022) 13:865748. doi: 10.3389/fendo.2022.865748 35634503 PMC9132262

[37] Zou Y Henry WS Ricq EL Graham ET Phadnis VV Maretich P. Plasticity of ether lipids promotes ferroptosis susceptibility and evasion. Nature. (2020) 585:603–8. doi: 10.1038/s41586-020-2732-8 PMC8051864 32939090

[38] Fernández-Real JM López-Bermejo A Ricart W. Cross-talk between iron metabolism and diabetes. Diabetes. (2002) 51:2348–54. doi: 10.2337/diabetes.51.8.2348 12145144

[39] Escobar-Morreale HF. Iron metabolism and the polycystic ovary syndrome. Trends Endocrinol Metab. (2012) 23:509–15. doi: 10.1016/j.tem.2012.04.003 22579050

[40] Tan W Dai F Yang D Deng Z Gu R Zhao X. MiR-93-5p promotes granulosa cell apoptosis and ferroptosis by the NF-kB signaling pathway in polycystic ovary syndrome. Front Immunol. (2022) 13:967151. doi: 10.3389/fimmu.2022.967151 36341347 PMC9626535

[41] Shi Q Liu R Chen L. Ferroptosis inhibitor ferrostatin-1 alleviates homocysteine-induced ovarian granulosa cell injury by regulating TET activity and DNA methylation. Mol Med Rep. (2022) 25:130. doi: 10.3892/mmr 35169856 PMC8867468

[42] Zhang L Wang F Li D Yan Y Wang H. Transferrin receptor-mediated reactive oxygen species promotes ferroptosis of KGN cells via regulating NADPH oxidase 1/PTEN induced kinase 1/Acyl-CoA synthetase long chain family member 4 signaling. Bioengineered. (2021) 12:4983–94. doi: 10.1080/21655979.2021.1956403 PMC8806504 34369274

[43] Tang H Jiang X Hua Y Li H Zhu C Hao X. NEDD4L facilitates granulosa cell ferroptosis by promoting GPX4 ubiquitination and degradation. Endocr Connect. (2023) 12:e220459. doi: 10.1530/EC-22-0459 36662677 PMC10083675

[44] Liu H Xie J Fan L Xia Y Peng X Zhou J. Cryptotanshinone Protects against PCOS-Induced Damage of Ovarian Tissue via Regulating Oxidative Stress, Mitochondrial Membrane Potential, Inflammation, and Apoptosis via Regulating Ferroptosis. Oxid Med Cell Longev. (2022) 2022:8011850. doi: 10.1155/2022/8011850 35419170 PMC9001078

[45] Peng Q Chen X Liang X Ouyang J Wang Q Ren S. Metformin improves polycystic ovary syndrome in mice by inhibiting ovarian ferroptosis. Front Endocrinol (Lausanne). (2023) 14:1070264. doi: 10.3389/fendo.2023.1070264 36755918 PMC9900736

[46] Zhang P Pan Y Wu S He Y Wang J Chen L. *n*-3 PUFA promotes ferroptosis in PCOS GCs by inhibiting YAP1 through activation of the hippo pathway. Nutrients. (2023) 15:1927. doi: 10.3390/nu15081927 37111146 PMC10145554

[47] Jiang Y Yang J Du K Luo K Yuan X Hua F. 1,25-Dihydroxyvitamin D3 alleviates hyperandrogen-induced ferroptosis in KGN cells. Hormones (Athens). (2023) 22:273–80. doi: 10.1007/s42000-023-00439-5 PMC10209298 36884209

[48] Hossain MS Mineno K Katafuchi T. Neuronal orphan G-protein coupled receptor proteins mediate plasmalogens-induced activation of ERK and akt signaling. PLoS One. (2016) 11:e0150846. doi: 10.1371/journal.pone.0150846 26934370 PMC4775022

[49] Ferreira da Silva T Granadeiro LS Bessa-Neto D Luz LL Safronov BV Brites P. Plasmalogens regulate the AKT-ULK1 signaling pathway to control the position of the axon initial segment. Prog Neurobiol. (2021) 205:102123. doi: 10.1016/j.pneurobio.2021.102123 34302896

[50] Che H Zhang L Ding L Xie W Jiang X Xue C. EPA-enriched ethanolamine plasmalogen and EPA-enriched phosphatidylethanolamine enhance BDNF/TrkB/CREB signaling and inhibit neuronal apoptosis *in vitro* and *in vivo* . Food Funct. (2020) 11:1729–39. doi: 10.1039/C9FO02323B 32043504

[51] Hossain MS Mawatari S Fujino T. Plasmalogens, the vinyl ether-linked glycerophospholipids, enhance learning and memory by regulating brain-derived neurotrophic factor. Front Cell Dev Biol. (2022) 10:828282. doi: 10.3389/fcell.2022.828282 35223852 PMC8864319

[52] Honsho M Mawatari S Fujino T. Transient Ca^2+^ entry by plasmalogen-mediated activation of receptor potential cation channel promotes AMPK activity. Front Mol Biosci. (2022) 9:1008626. doi: 10.3389/fmolb.2022.1008626 36406270 PMC9672372

